# Outcomes in a Cohort of Patients Started on Antiretroviral Treatment and Followed up for a Decade in an Urban Clinic in Uganda

**DOI:** 10.1371/journal.pone.0142722

**Published:** 2015-12-07

**Authors:** Barbara Castelnuovo, Agnes Kiragga, Joseph Musaazi, Joseph Sempa, Frank Mubiru, Jane Wanyama, Bonnie Wandera, Moses Robert Kamya, Andrew Kambugu

**Affiliations:** 1 Infectious Diseases Institute, Makerere University, Mulago Hospital, Kampala, Uganda; 2 School of medicine, Makerere University, Mulago Hospital, Kampala, Uganda; Rush University, UNITED STATES

## Abstract

**Background:**

Short-medium term studies from sub-Saharan Africa show that, despite high early mortality, substantial loss to program, and high rates toxicity, patients on antiretroviral treatment have achieved outcomes comparable to those in developed settings. However, these studies were unable to account for long term outcomes of patients as they stayed longer on treatment.

**Objectives:**

We aim to describe ten years outcomes of one of the first cohort of HIV positive patients started on antiretroviral treatment (ART) in Sub-Saharan Africa.

**Methods:**

We report 10-years outcomes including mortality, retention, CD4-count response, virological outcomes, ART regimens change from a prospective cohort of 559 patients initiating ART and followed up for 10 years Uganda.

**Results:**

Of 559 patients, 69.1% were female, median age (IQR) was 38 (33–44) years, median CD4-count (IQR) 98 (21–163) cell/μL; 74% were started on stavudine, lamivudine and nevirapine, 26% on zidovudine, lamivudine and efavirenz. After 10 years 361 (65%) patients were still in the study; 127 (22.7%) had died; 30 (5%) were lost to follow-up; 27 (5%) transferred; 18 (3%) withdrew consent. The probability of death was high in the first year (0.15, 95%, CI 0.12–0.18). The median CD4 count increased from 98 to 589 cell/μL (IQR: 450–739 cell/μL) with a median increase of 357 cells/μL (IQR: 128–600 cells/μL); 7.4% never attained initial viral suppression and of those who did 31.7% experienced viral failure. Three hundred and two patients had at least one drug substitution while on first line after a median of 40 months; 66 (11.9%) of the patients were switched to a second line PI-based regimen due to confirmed treatment failure.

**Conclusions:**

Despite the high rate of early mortality due to advanced disease at presentation the outcomes from this cohort are encouraging, particularly the remarkable and incremental immune-recovery and a satisfactory rate of virologic suppression.

## Introduction

Over 7.6 million individuals are receiving antiretroviral therapy (ART) in sub-Saharan Africa (SSA) as a result of unprecedented global health initiatives to scale-up ART delivery since 2003[1]. Uganda was one of the first countries to roll-out public-sector HIV and ART programs on a large scale, commencing in 2004[2]. These efforts were mainly supported and funded by the World Health Organization (WHO), Global Fund to Fight AIDS, Tuberculosis and Malaria (GFATM), and the United States President’s Emergency Plan for AIDS Relief (PEPFAR). After an initial roll out in national and regional referral hospitals, access to antiretroviral treatment was gradually expanded to peripheral facilities with more than 400,000 HIV positive individuals receiving ART by the end of 2012 [3].

Short and medium term studies from sub-Saharan Africa show that despite high early mortality rates due to late presentation[4], substantial loss to program[5], and high rates of drug substitution due to toxicity [6], antiretroviral treatment programs have achieved outcomes comparable to those in developed settings especially in terms of CD4 count gains [7] and viral suppression[8]. Furthermore adherence levels in sub-Saharan Africa, once a big concern before the scale up of ART, have been reported to be similar or higher than those achieved by patients in resource-rich settings [9]. However, these studies were done for a short duration and were unable to account for long term outcomes of patients as they stayed longer on treatment and as their well -being improved.

The Infectious Diseases Institute at the Makerere University College of Health Sciences in Kampala was one of the first HIV care centers to receive free antiretroviral drugs from GFATM and PEPFAR, therefore some of the active patients attending the adult clinic have been followed up for more than ten years. We have previously described the short term (one-year) and medium term (four-years) outcomes from a well characterized ART cohort within a large urban HIV treatment center at the National Referral Hospital in Uganda [10]. The objective of this analysis was further to evaluate the long term outcomes in this cohort of patients initiated on ART at the beginning of free treatment roll out in Uganda, and followed up for a decade. Specifically we assessed four outcome indicators: retention in care, virologic suppression and immune-recovery, ART regimen changes.

## Methods

### Ethical statement

The study was reviewed and approved by the Makerere University Faculty of Medicine Research and Ethics Committee (Approval number: 016–2004) and the Uganda National Council for Science and Technology (Approval number: MV 853). All patients provided written informed consent.

### Study site and population

The Infectious Diseases Institute (IDI) is a large urban HIV center of excellence [11] located at Mulago National Referral Hospital in Kampala, Uganda. IDI began providing free antiretroviral treatment through the GFATM and PEPFAR since April 2004. At present more than 30,000 patients have been registered and over 15,000 have ever been started on ART. ART efficacy is monitored through bi-annual CD4 counts and viral load measurements were performed ad hoc in patients with immunologic failure according to the WHO guidelines [12]; ART toxicity was not routinely monitored but safety laboratory test were performed upon clinician’s discretion.

In this context a cohort of consecutive adult patients starting ART was initiated and 559 patients were enrolled in a research cohort between April 2004 and April 2005. The main objective of this study, hence forth referred to as the IDI Research Cohort, was to assemble and to characterize a cohort of patients on ART in order to address pertinent epidemiologic, clinical and psychosocial research questions relevant to the national and sub-Saharan African ART roll-out.

### Study procedures

At enrollment and every three months patients were evaluated by a dedicated study clinician and counselor. ART was started according to WHO and Uganda Ministry of Health guidelines [13,14] in patients with an AIDS defining illness or a CD4 count <200 cell/μ with a combination of stavudine (weight-adjusted), lamivudine and nevirapine or zidovudine, lamivudine and efavirenz.

Detailed study procedures for this is cohort are described elsewhere[10], but in summary: at enrollment and during the follow up study visits information was collected on patients’ demographic, previous and current opportunistic infections as well as non-HIV related clinical events, WHO staging, vital signs and ART regimen, and physical examination was performed. During the follow up visits adherence was assessed using the visual analog scale and ART toxicity and reasons for ART substitution were recorded. Treatment regimen was changed with a single drug in patients who experienced a grade 3 or 4 toxicity according to the AIDS Clinical Trials Group (ACTG) classification. Efavirenz was substituted with nevirapine (up to 2012) in women found to or those planning to become pregnant, while efavirenz was substituted with nevirapine among patients diagnosed with tuberculosis. Patients with 2 consecutive viral loads greater than 1000 copies/ml were eligible for switch to protease inhibitors (PIs) based regimens if second line drugs were available. Patients with a first viral load greater than 400 copies/ml were offered extra adherence counselling. However, in our context, where second line drugs are not always readily available, it is not uncommon that patients with detectable viral load but low levels of viremia are maintained on failing first line regimens, with additional adherence counseling.

Laboratory investigations were performed every 6 months and these included complete blood cell count, liver and renal function tests, CD4 count by FACSCount (Becton Dickinson, San Jose, CA and, more recently, by FACSCalibur, Becton Dickinson), HIV viral load by Amplicor HIV-1 Monitor PCR Test version 1.5 and more recently, COBAS Ampliprep/COBAS Taqman HIV-1 Test Ver.2.0 (Roche Diagnostics, Indianapolis, IN) and storage of 5 ml of plasma at -80 C for future testing. Laboratory testing was performed at the Makerere University–Johns Hopkins University Core Laboratory, which is certified by the College of American Pathologists.

Tracking and complete ascertainment of patients who did not attend their study appointment was carried out in real time by phone call and/or home visit. In case of death the cause was determined from a combination of source documents and a structured interview with the patient’s next of kin (“verbal autopsy”). Generally ascertainment of outcomes of nearly all study subjects was achieved and lost to follow up was negligible.

### Data collection and data quality

Data was collected on a study specific designed questionnaire by the study counselor and the study clinician. The data was entered into a database designed using Oracle up to year 2011 and in ICEA (Integrated Clinic Enterprise Application), an application developed by a team of software developers based at the IDI, which ensures good quality of collected data [15].

The entered data was subsequently validated by a senior data entrant before being approved. Laboratory results were obtained directly from the laboratory database. The data was also regularly checked for quality, completeness and discrepancies by a data quality assurance team and a data manager.

### Definitions

A patient was considered *retained* in the cohort if he/she was alive and still enrolled in the cohort. A patient was defined as *lost to follow up* if two consecutive study visits were missed, i.e. the patient did not attend the clinic for more than 6 months.

*Viral suppression* was defined as the attainment of HIV viral load <400 copies/ml after starting ART in patients with at least 6 months of follow up, and in those with documented viral suppression, *viral failure* was defined as 2 consecutive viral load measurement >400 copies/ml or 1 viral load> 5,000 copies/ml if a subsequent viral load was not available. *Treatment failure* was defined as the lack of attainment of viral suppression or the occurrence of viral failure after initial suppression.

*Treatment change* was defined as the occurrence of drug substitution or regimen switch. *Drug substitution* was defined as the modification of at least one drug within first line regimen, while *regimen switch* was defined as a change from a first to a second line regimen.

### Statistical analysis

Baseline characteristics were described using medians and proportions and stratified by gender. Gender categories were compared using chi-square test for categorical variables and Wilcoxon rank-sum test for not normally distributed variables.

To describe retention in the cohort we reported the number and proportion of patients who were no longer enrolled in the cohort for any reason including loss to follow up, death, transfer out, and withdrawal of consent at month 6, 12, and year 2, 5 and 10. We reported mortality over a 10-year time period by cause: HIV related, other medical, and accidental and we calculated the probability of death using Kaplan Meier estimates with right censoring at time of death, stratified by gender and CD4 count μL at ART start (<50 cells/μL, 50–99 cells/μL, and ≥100 cells).

We described the increase in CD4 count from baseline to all subsequent 6-monthly CD4 count readings and the overall increase from baseline to the last visit in the cohort. We also reported the proportion of patients at each time point with a CD4 count >400 cells/μL, which is the lower normal limit of CD4 count in Ugandan adults [16].

We reported the proportion of patients who attained viral suppression and of those who experienced viral failure. We also calculated the cumulative probability of treatment failure stratified by gender and CD4 count at ART start at different thresholds.

We described the ART regimens prescribed during the 10 years categorized as stavudine, zidovudine tenofovir based for first line regimens, and PI based. We described all causes of drug substitution categorized as toxicity, treatment failure, new episode of tuberculosis, pregnancy/intended pregnancy and others, and we calculated the yearly and cumulative probability of first drug substitution for any reason while of first line.

All differences in survival probabilities were compared using the log rank test.

The analysis was performed using STATA® version 12.2, Texas USA.

## Results

### Baseline characteristics

Between April 2004 and April 2005, 559 consecutive patients starting first line antiretroviral treatment were enrolled in the IDI Research Cohort. The baseline characteristics and the characteristics by gender are summarized and compared in Table 1. The majority (69%) of the patients was female, in WHO clinical stages 3 and 4 (89%), with a median CD4 count of 98 cells/μL (IQR: 21–163). The characteristics of the patients in the cohort were similar to the characteristics of other patients started on ART at IDI but not enrolled in the study [17]. Overall male patients were older, and presented with more advanced WHO stage, had lower body mass index (BMI) and median CD4 count; a higher proportion of male was started on efavirenz-based regimen due to concurrent tuberculosis (TB) treatment or being under investigation to rule out TB (Table 1).

**Table 1:** Baseline characteristics stratified by gender of the patients enrolled in the IDI Research Cohort.

Characteristics	Total N = 559	Females N = 386 (69%)	Males N = 173 (31%)	P value
**Age in years, median (IQR)**	38 (33–44)	34 (29–40)	38 (33–42)	0.00
**WHO clinical stage 3 and 4**	496 (89%)	339 (87.8%)	158 (91.3)	0.22
**BMI (Kg/m** ^**2**^ **), median (IQR)**	20 (17–22)	20.3 (18.2–22.7)	19.4 (17.5–21.0)	0.00
**Hb g/dL, median (IQR)**	11.5 (10.3–12.9)	11.2 (10.0–12.5)	12.6 (11.0–14.1)	0.00
**CD4 count cell/μL, median (IQR)**	98 (21–163)	100 (29–170)	87 (13–151)	0.09
**HIV RNA log copies/ml, median (IQR)**	5.4 (5.0–5.8)	5.4 (5.0–5.8)	5.5 (5.2–5.7)	0.27
**ART Regimen d4T+3TC+ nevirapine**	414 (74%)	294 (76.2%)	120 (69.4%)	0.09
**AZT+3TC+ efavirenz**	145 (26%)	92 (23.8%)	53 (30.6%	

WHO: world Health Organization; BMI: body mass index; Hb: hemoglobin; ART: antiretroviral treatment; d4T: stavudine; 3TC: lamivudine; AZT: zidovudine

### Survival and retention into care

**Fig 1:**
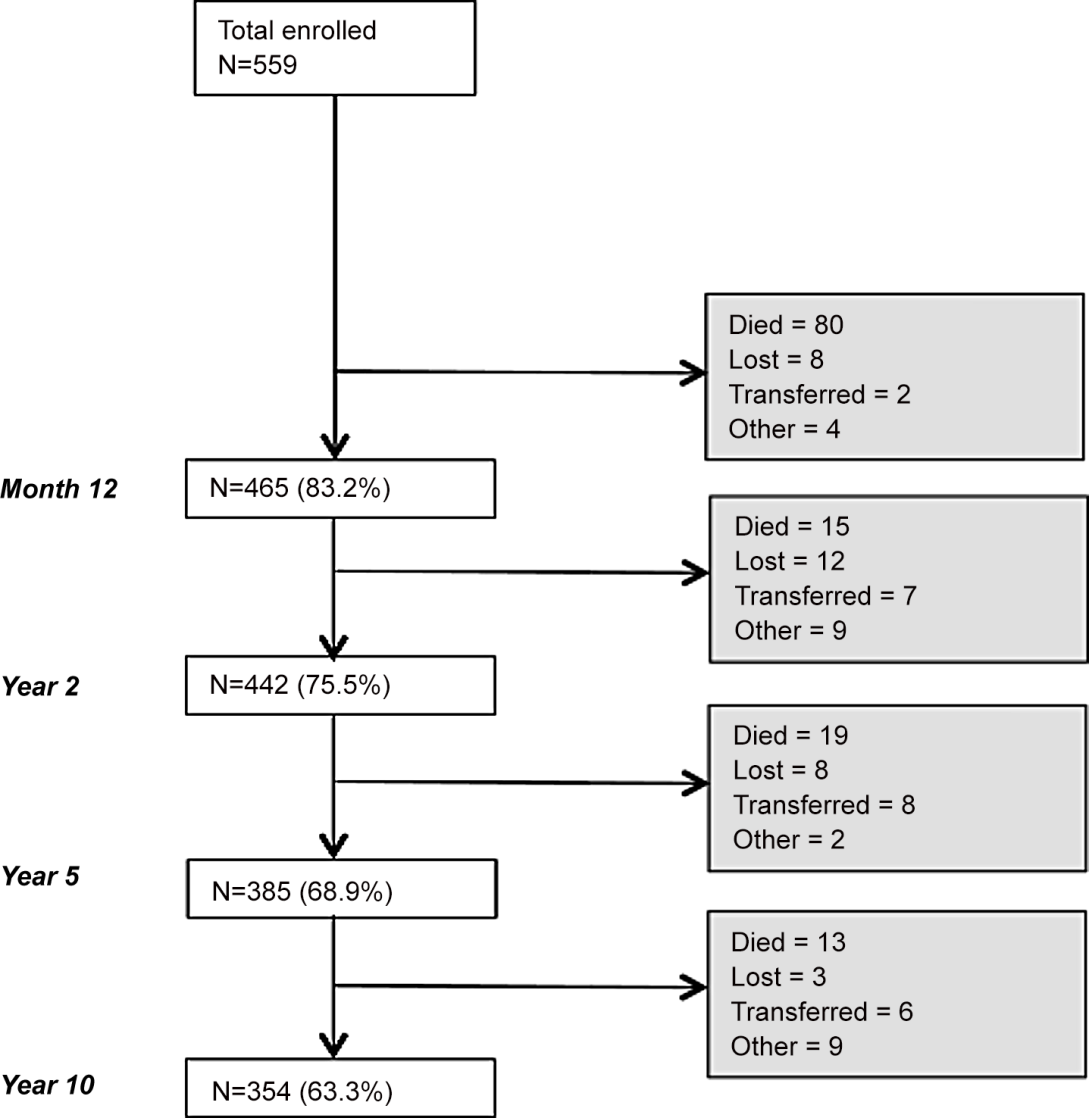
Retention in the IDI Research Cohort.

The median follow up time was 110 months (IQR: 25–111). Fig 1 shows the follow up status of the patients during the follow up. Overall 127/559 patients (22.7%) died. of which 63% died during the first year on ART, and 31 (5.5%) were lost to follow up. The probability of being retained in the study was 0.81 (95%CI: 0.77–0.84) by year 1, 0.70 (95%CI: 0.66–0.73) by year 5, and 0.64 (95%CI: 0.60–0.68) by 10 years on ART.

The probability of death was 0.15 (95%CI: 0.12–0.18) by year 1, 0.21 (95%CI: 0.18–0.25) by year 5, and 0.24 (95%CI: 0.21–0.28) by 10 years on ART. The majority (86, 67.7%) of the deaths were AIDS events, followed by other medical condition (24, 18,9%) and 1 (0.8%) accidental death; the cause of death could not be determined for 16 (12.6%) patients due to lack of relative contact or non-conclusive “verbal autopsy”. Patients with a CD4 count <50 cells/μL at ART start had a significant higher overall probability of dying, 0.31 (95%CI: 0.25–0.39) as compared to patients with a CD4 count ≥100 cells/μL, 0.18: (95%CI: 0.14–0.23) (P value 0.001). We did not find an independent association between survival and gender (P value 0.7).

### Immune-recovery

**Fig 2:**
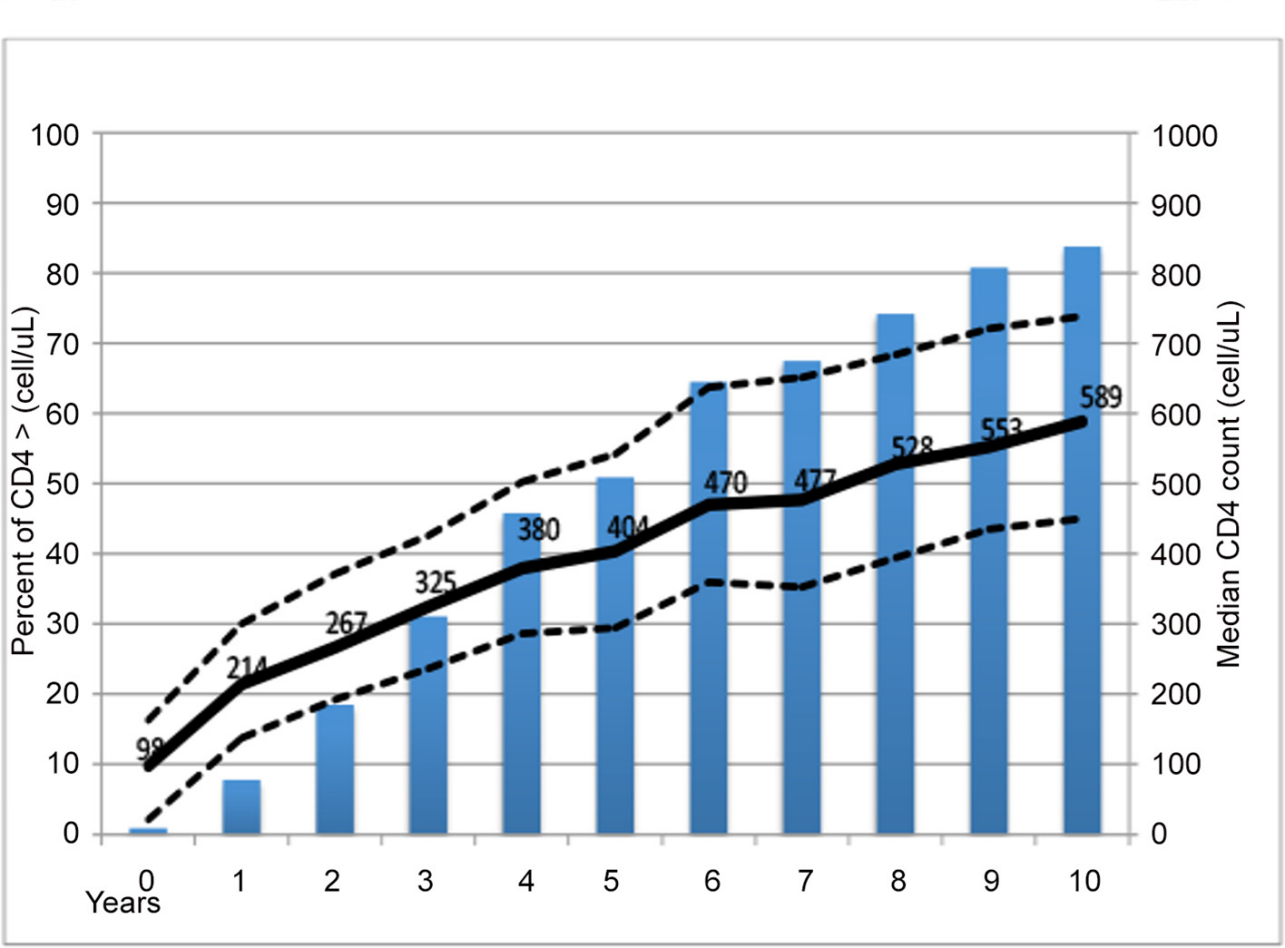
Trends in median CD4 count and proportion of patients with CD4 count > 400 cells/μL over 10 years of follow up on ART.

The median CD4 count increased from 98 to 589 cell/μL (IQR: 450–739 cell/μL) over the 10 year period with a median increase of 357 cells/μL (IQR: 128–600 cells/μL). The proportion of patients with a CD4 count >400 cells/μL rose from 0.9% at baseline to 83.8% after ten years on ART, with majority of patients (50.9%) being above this threshold by year 5 on ART (Fig 2). The cumulative probability of attaining a CD4 count >400 cells/μL was 0.17 (95%CI: 0.14–0.21) at year 1, 0.72 (95%CI: 0.68–0.77) at year 5, and 0.92 (95%CI: 0.89–0.94) at year 10 on ART.

### Treatment failure

Four hundred seventy four patients were followed up for at least 6 months, and of these 35 (7.4%) never attained viral suppression; the cumulative probability of attaining viral suppression was 0.94 (95% CI: 0.92–0.96). Among the 439 (92.6%) patients who attained viral suppression 139 (31.7%) experienced subsequent viral failure with a cumulative probability of 0.34 (95%CI: 0.30–0.39). Five of those who never attained viral suppression, and 14 and those who experienced viral failure subsequently died.

Overall the median time to treatment failure was 30 months (IQR 11–66) and the cumulative probability of treatment failure over 10 years was 0.38 (0.34–0.43).The probability of treatment failure was similar in males as compared to females (0.37, CI 0.32–0.43 versus 0.45, CI 0.36–0.53), P = 0.08) and we did not find any difference in the probability of treatment failure across the CD4 count strata at ART start (P = 0.52)

### Treatment regimens and treatment change

**Fig 3:**
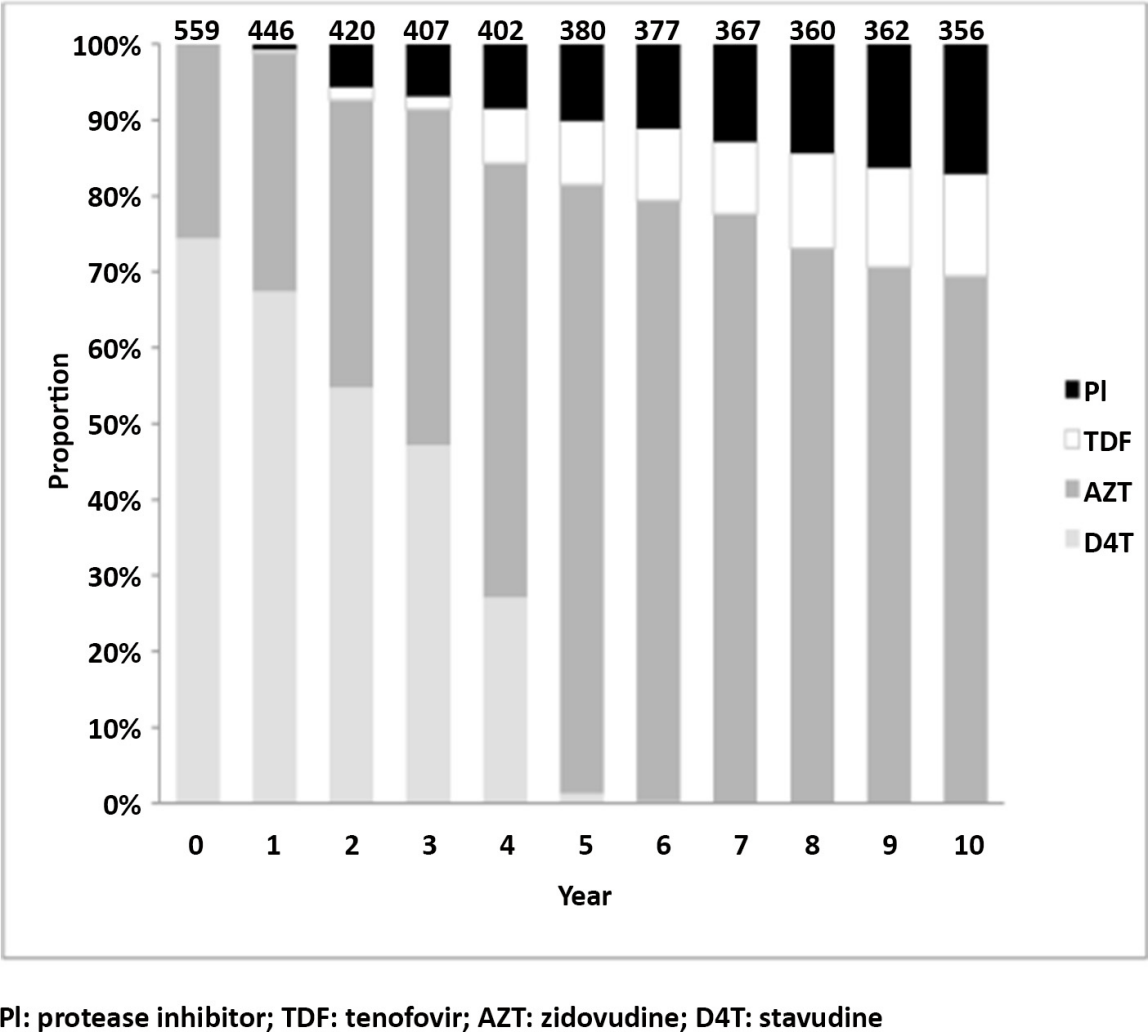
Proportions of antiretroviral treatment regimens received by the patients during 10 years of follow up.

Fig 3 shows the proportion of treatment regimens received by the patients in the cohort during the 10 years of follow up. While the majority (74.4%) of patients were started on stavudine based regimens, after 7 years of follow up none of the patients was receiving stavudine; conversely the proportion of patients receiving zidovudine increased from 15.6% to 69.4% and of those receiving tenofovir from 0% to 13.5%; in the 10^th^ year of follow up 17.1% of the retained patients were switched to second line PI-based regimens.

Three hundred and two patients (54%) had at least one drug substitution while on first line for a total of 378 drug substitutions. Two hindered forty four (64.6%) had at least one drug substitution, 46 (24.3%) two substitution and the remaining ones up to 5 substitutions. The most common reason (141, 46.7%) for the first drug substitution was the 2008 Ministry of Health recommendation to replace stavudine with a less toxic nucleoside reverse transcriptase inhibitor in patients still receiving it, followed by ART toxicity (115, 32.9%), occurrence of a new episode of tuberculosis (24, 8%), pregnancy (14, 4.6%), and other reasons (8. 2.6%).

**Fig 4:**
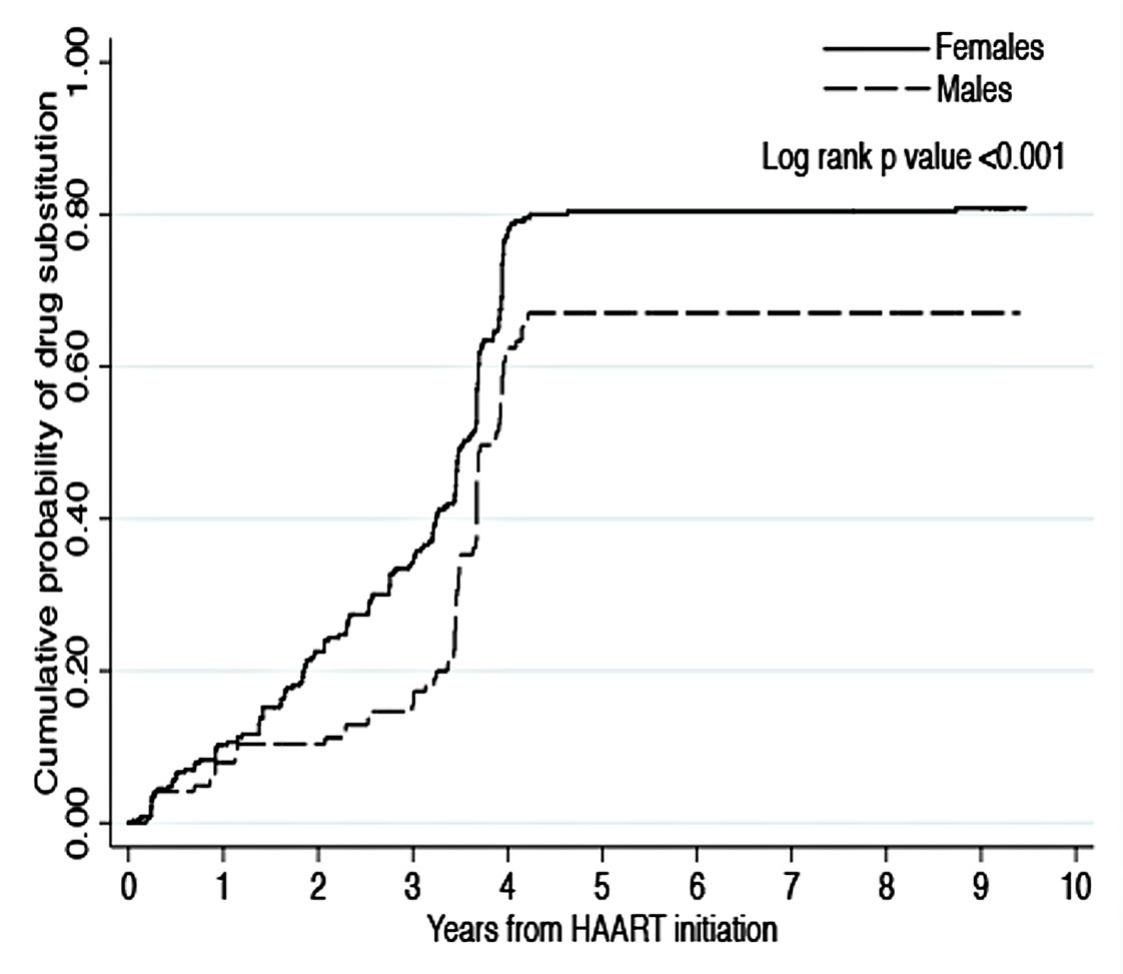
Cumulative probability of drug substitution by gender over 10 years of follow up.

The median time to the first drug substitution was 40 months (20–44); the cumulative probability of drug substitution was 0.77 (CI: 0.72–0.81) with a higher probability of substitution in females (0.81, CI: 0.76–0.85) as compared to males (0.67, CI 0.58–0.76, P = <0.001) (Fig 4).

Overall 66 (11.9%) of the patients were switched to a second line PI-based regimen due to confirmed treatment failure.

## Discussion

To our knowledge this is the first comprehensive analysis of 10-year outcomes in a well characterized African ART cohort. We observed that despite a high early mortality, the overall retention and viral suppression rates were high with remarkable immunologic recovery over the 10 year period. These findings are encouraging given the context of an initially highly immune-deficient population. Unfortunately this success was overshadowed by high rates of early mortality due to late presentation and high rate of toxicity resulting in drug substitution.

Overall we observed that our cohort consisted of young, predominantly female and severely immunosuppressed individuals, representative of African ART patients at the start of the ART roll-out. The population in this cohort started ART at very advanced stage of disease, with 89% of the patients being in 3 or 4 WHO stages, and with a quarter of them having a CD4 count <21 cells/μL. Male patients were generally older and had a lower BMI probably due to a later presentation into HIV care.

The overall rate of loss to follow up was very low, but this may not reflect the attrition rates observed in other settings [18], or even for patients routinely followed up in our adult clinic, but not enrolled in a research study. The patients enrolled in this cohort had initial and ongoing intensive counseling, they were reminded the day before the study visit about their appointment by phone call, and were systematically traced if the appointment was missed, or visited at home if the attempt of contacting them was unsuccessful. Moreover patients in this cohort had 6 monthly viral load measurements with review of these results with the patient at subsequent visits. The reported level of retention may not be achieved in routine busy settings where resources are minimal and health care workers are overwhelmed, but we do demonstrate that with a dedicated clinical team and routine viral load monitoring high retention into care can be attained. The findings are at present even more encouraging with the current efforts to roll out routine viral load monitoring in resource limited settings, which in the Uganda case have commenced at the larger HIV treatment centers.

Despite a low baseline CD4 count, we report remarkable immune recovery (median CD4 count after 10 years: 589 cells/mL) with CD4 count levels continuously increasing throughout the 10 years follow up period up and with 83% of the patients attaining a CD4 count above the lower normal limit. The reduction in incidence of OIs including tuberculosis (data not shown) and mortality is to a large extent attributable to this immune recovery. In this analysis we included all patients regardless of viral suppression and ART regimen in order to demonstrate the general effect of ART on population immune recovery over time.

We also observed a high initial viral response comparable or higher than observed in on treatment analysis from similar African [19] and non-African [20] settings with only 7% of the patients not achieving viral suppression; it is likely that in this cohort acquired HIV resistance was extremely low due to limited number of patients on ART in Uganda before 2004, and therefore the lack of achievement of suppression should be mostly attributed to early suboptimal adherence. Thirty two percent of the patients with viral suppression experienced subsequent viral failure; our results may not be comparable with others from sub-Saharan Africa due to the longer follow up and variation of definition of viral failure[19]. Similarly to what was observed in a cohort from similar settings [21] the probability of treatment failure rose more sharply during the first 2 years on ART as compared to subsequent follow up period. Viral suppression is associated with reduction in OI incidence, as well as non-communicable diseases-related to the markedly reduced immune-activation. This observation of high viral suppression is additionally important from the perspective of HIV prevention and underscores the gains of treatment as prevention (TasP).

Similarly to other reports of patients started on ART at the beginning of the scale up [4,22], a high proportion of patients died. Seventy five percent of the deaths occurred within two years of follow up and sixty three percent during the first year; the majority of the deaths were HIV related and associated with low CD4 count at ART start suggesting that those patients died as result of late presentation with underlying opportunistic infections, rather than inadequate quality of care.

More than half of the patients required at least one drug substitution while on first line; this was mostly due to the inclusion of stavudine in the majority of the ART regimens prescribed at treatment start. Most of the substitutions due to toxicity were attributable to stavudine toxicity [23] while the most common reason for drug substitution was the Ministry of Health recommendation to phase out stavudine from the ART national program; the majority of the patients subsequently received zidovudine since tenofovir is more expensive and was reserved for patients intolerant to zidovudine. Similarly to other studies women had a higher probability of having drug substitution, mostly due to stavudine toxicity [6,8].

In summary, although the success of ART was jeopardized in the early years by early mortality and ART toxicity, the outcomes from this cohort are encouraging, particularly the remarkable and incremental immune-recovery and a satisfactory rate of virologic suppression. Patients presenting with less advanced disease and those started on ART later during the scale up when stavudine was no longer part of the preferred regimens are likely to experience even more favorable outcomes.
